# Intraarticular vancomycin powder is effective in preventing infections following total hip and knee arthroplasty

**DOI:** 10.1038/s41598-020-69958-0

**Published:** 2020-08-03

**Authors:** Georg Matziolis, Steffen Brodt, Sabrina Böhle, Julia Kirschberg, Benjamin Jacob, Eric Röhner

**Affiliations:** Orthopaedic Department Waldkliniken Eisenberg, Orthopaedic Professorship of the University Hospital Jena, Klosterlausnitzer Str. 81, 07607 Eisenberg, Germany

**Keywords:** Orthopaedics, Outcomes research

## Abstract

Locally applied vancomycin is increasingly being used in primary hip and knee arthroplasty to reduce the risk of infection. Despite encouraging initial results, considerable debate remains on the basis of the data currently available. In particular, it has been unclear up to now whether local vancomycin is suitable to further reduce the risk of infection even if the rate of infection is already low (< 1%). In this monocentric retrospective cohort study, all primary total hip and knee arthroplasties performed between 2013 and 2018 were included. After a change in procedure at the hospital, 1 g vancomycin powder was applied intraarticularly before wound closure. The remaining perioperative procedure was constant over the investigation period. The follow-up was one year. The presence of an infection according to the currently valid MSIS criteria was defined as the endpoint. In patients with TKA two infections (0.3%) were observed under vancomycin prophylaxis in contrast to 44 infections (1.3%) in the control group (*p* = 0.033). In patients with THA two infections (0.5%) were observed under vancomycin prophylaxis and 48 infections (1.1%) in the control group without local vancomycin but this difference was statistically not significant. No wound complications requiring revision were observed as a result of the vancomycin. On the basis of the results of this study, intraarticular application of vancomycin powder in total hip and knee arthroplasty may be considered. Prospective randomized studies have to confirm this promising results prior a common recommendation.

*Level of Evidence III* Retrospective cohort study.

## Introduction

Infections after endoprosthetic hip or knee replacement, in spite of all perioperative improvements, are observed with an incidence of 1–2%^1–4^. Despite the apparently low risk, efforts to further reduce the risk are sensible in light of the consequences for the patients concerned, with the potential for several follow-up operations and the often limited functional outcome.

In spinal surgery, the reduction in the rate of infection through locally applied vancomycin was established more than 10 years ago and has been well documented by meta-analyses and animal experiments^5–8^. The positive effect is set against minor complications caused in 0.3% of cases, mostly culture-negative seroma formation^9^^,^ so that it is even used successfully in children^10^.

Otte et al. were able to show that intrawound vancomycin powder lowers the infection rate after knee and hip arthroplasty from 1.6 to 0.5%, compared with a control group^11^. In a subgroup analysis, the effect in revision operations was even greater, with a reduction of the infection rate from 3.9 to 0%. Patel et al. showed a reduction in the infection rate through vancomycin of 2.7–0.3% and determined a number needed to treat (NNT) of 47.5^12^. Systemic side effects were not observed. The local application of vancomycin was assessed to be safe and cost-effective.

Dial et al. observed a similar effect in primary hip arthroplasty, with a lowering of the infection rate from 5.5% without vancomycin to 0.7% with intrawound vancomycin powder^13^. However, sterile wound complications were seen in 4.4% of the cases treated with vancomycin, compared with 0% in the control group. Hanada et al. had sterile wound complications in as many as 11.8% of cases after vancomycin, compared with 4.3% in the control group^14^. This paper failed to show a statistically significant advantage of the intraoperative administration of vancomycin, so that the authors did not recommend its use.

In a current meta-analysis, pooled data were used to demonstrate a significant reduction of the infection rate through locally applied vancomycin, with an odds ratio of 0.44^15^.

Also, in the implant-retaining treatment of early infection by irrigation, debridement and exchange of all modular components, it was shown that a combination of povidone-iodine and vancomycin increases the success rate of this procedure^16^.

In the studies that demonstrated a positive effect, however, the infection rate was between 1.6 and 5.5%, and was thus higher than the incidence of less than 1% described for many centers with optimized perioperative prophylaxis^17–21^. It is unclear whether locally applied vancomycin can also lower the risk of infection in an optimized setting with an already low infection rate.

Up to now, there has been a lack of monocentric studies with sufficiently high case numbers to demonstrate the efficacy of vancomycin when there is already a low infection rate of less than 1%. This requires considerably higher case numbers than those published to date, with constant perioperative management. Therefore, the hypothesis of the present study is that locally applied vancomycin can also further reduce an already low risk of infection.

## Methods

This is a monocentric retrospective cohort study that is designed to investigate the effect of a change in the orthopaedic department’s practice, i.e., the intraoperative administration of 1 g intraarticular vancomycin powder in total hip and knee arthroplasty. It was not used selectively, so that an inclusion bias due to restricted use in specific indications could be excluded. The study was approved by the ethics committee of the University Hospital Jena (2019–1395). Resulting from the retrospective and anonymized study design, an informed consent was not necessary according to the ethics committee approval. All methods were carried out in accordance with the relevant guidelines and regulations based on the approval.

All primary total knee and hip arthroplasties performed between 2013 and 2018 due to primary or secondary osteoarthritis were included. Cases of postinfectious osteoarthritis as well as revision surgery were excluded, in order to prevent falsification through a potentially already existing infection or contamination.

The follow-up time was defined to one year for all patients. This was done to avoid higher infection rates in patients with a longer follow up resulting from bacteremia unrelated to the operation. The presence of an infection was defined as the endpoint. This was determined on the basis of the medical files of patients returning for follow-up examinations or readmissions within one year after operation at our hospital for any cause.

Except for the intraarticular application of vancomycin, the perioperative regimen was constant for the whole study period and did not differ between the two study groups.

All patients were washed with antiseptic (Stellisept, Hartmann, Heidenheim, Germany) one day before the operation. No preoperative instructions for bathing or at home cleansing process was used. This skin was not shaved, and hair was trimmed immediately prior to the operation only in the event of interfering hair growth (3M Surgical Clipper, Saint Paul, USA). Cutasept (Hartmann, Heidenheim, Germany), was used for disinfection. All total hip arthroplasties (THA) were covered with an iodine-impregnated incision drape (Ioban, 3M, Saint Paul, USA). In case of a known iodine allergy a drape without iodine was used. Total knee arthroplasties (TKA) were covered without a drape. Single shot antibiotic prophylaxis was applied 30 min prior to skin incision (2 g cefazolin). In case of an allergy, clindamycin was administered. If the operation lasted longer than 2 h, the antibiotic prophylaxis was repeated once.

Patients were included via a database search for procedures 5-820 (THA) and 5-822 (TKA) performed between 2013 and 2018. Surgery documentation revealed whether or not intraarticular vancomycin powder (1 g) was administered. A further database search identified all patients who were readmitted for any reason after prosthesis implantation. The records were screened for the presence of an implant associated infection. The diagnosis was confirmed according to the available microbiological laboratory findings and surgery reports on the basis of current MSIS criteria (Table 1)^22^.

**Table 1:** Adopted MSIS criteria according to Shohat et al.^22^.

	Threshold	Score
**Major criteria**		
Two positive growth of the same organism (standard culture)		Yes = infection
Sinus tract with evidence of communication to the joint		Yes = infection
**Minor criteria**		
Serum CrP	> 10 mg/l	2
Synovial WBC	> 3.000 cells/µl	3
Synovial PMN	> 70%	2
Single positive culture		2
Positive histology		3
Intraoperative purulence		3
		Sum > 5 = infection

In addition, noninfectious wound problems were recorded. Such a wound revision was recorded as a potential complication of vancomycin powder usage.

All infected cases were treated according to the latest guidelines of the Infectious Diseases Society of America (IDSA)^23,24^. The IDSA is a medical association of health care professionals who specialize in infectious diseases and recommend diagnosis and treatment algorithms based on the actual evidence.

### Statistics

The group comparison was conducted with regard to the primary endpoint of infection using the chi-squared test, while all other demographic comparisons were tested for differences using the t-test. All statistical tests were conducted at a significance level of 0.05. A post-hoc power analysis was performed.

## Results

Over 5 years, 8,945 joints were included in the study. Intraarticular vancomycin powder was used in 1,082 joints (432 THA, 650 TKA) and was not used in 7,863 joints (4,392 THA, 3,471 TKA). In no case was an aseptic wound revision performed related to the application of vancomycin powder.

Regarding all total joint arthroplasties, four infections (0.4%) in the vancomycin group were observed in contrast to 92 infections in the control group without local vancomycin application (1.1%). This difference was statistically significant (*p* = 0.017, Table 2). Nine patients received a bilateral simultaneous procedure (4 THA and 5 TKA), all of them were in the control group without vancomycin and none of them was infected.

**Table 2:** Comparison of infection rates of TKA and THA, *p* = 0.017.

TKA and THA	Vancomycin	Control
Infection	4	92
No infection	1,078	7,771
Sum	1,082	7,863
Infection rate	0.4%	1.1%
Female:male ratio	1.6	1.5
Age (years)	69 ± 10	68 ± 9

In patients with TKA two infections (0.3%) were observed under vancomycin prophylaxis in contrast to 44 infections (1.3%) in the control group (*p* = 0.033, Table 3). In patients with THA two infections (0.5%) were observed under vancomycin prophylaxis and 48 infections (1.1%) in the control group without local vancomycin but this difference was statistically not significant (*p* = 0.216, Table 4). Based on the numbers the statistical power of the Chi^2^ test was calculated to be higher than 99% for every test.

**Table 3:** Comparison of infection rates of TKA, *p* = 0.033.

TKA	Vancomycin	Control
Infection	2	44
No infection	648	3,437
Sum	650	3,471
Infection rate (%)	0.3	1.3

**Table 4:** Comparison of infection rates of THA, *p* = 0.216.

THA	Vancomycin	Control
Infection	2	48
No infection	430	4,344
Sum	432	4,392
Infection rate (%)	0.5	1.1

Staph. epidermidis and staph. aureus were the most common bacteria in both groups (Table 5). Patients’ body mass index and ASA scores did not differ between the groups (Table 6).

**Table 5:** Bacterial growth in the vancomycin and control group.

Bacteria	Vancomycin	Control
Staph. epidermidis	2	26
Staph. aureus	2	15
Staph. capitis		6
Staph. haemolyticus		5
Staph. agalactiae		2
Staph. hominis		1
Enterococcae		6
*Escherichia coli*		6
Streptococcae		4
*Peptinophilus asaccarolyticus*		1
Propionibacterium		2
*Proteus mirabilis*		1
*Pseudomonas fluorescens*		1
Culture negative infection		16

**Table 6:** Body mass index (BMI) and American Society of Anesthesiologists (ASA) score for each group.

	Vancomycin	Control
BMI (kg/m^2^)	29.5 ± 5.2 (12.9–49.1)	29.8 ± 5.4 (10.9–64.3)
ASA score	2.3 ± 0.5 (1–4)	2.2 ± 0.5 (1–4)

There was no significant difference.

## Discussion

The main result of the present study is that intraarticular vancomycin powder lowers the infection rate after total knee arthroplasty and possibly after total hip arthroplasty. At the same time, no local wound complications requiring revision were observed.

Both results contradict the data of Hanada et al., who observed local aseptic complications due to vancomycin powder without a reduction of the infection rate^14^. The infection rates of 7.6% (control group) and 4.5% (vancomycin group) described in this study are far higher than the 1–2% reported in the literature for primary arthroplasty. In addition, the number of cases is insufficient to exclude an effect of vancomycin, particularly since the infection rate in the vancomycin group is nominally lower than that in the control group.

The present study could be explained by the predominantly intraarticular application of vancomycin. The powder was placed in the joint before capsule closure and, after capsule closure, the powder adhering to the container was tapped into the subcutaneous tissue. Therefore, a high concentration of vancomycin was not to be expected in the immediate vicinity of the skin.

This is in line with a review of the use of vancomycin powder on the spine, which likewise did not detect an elevated rate of aseptic wound complications^9^.

An effect of vancomycin powder on wear in the sense of a potentially conceivable third-body wear was ruled out in vitro, so that long-term sequelae are improbable^25^.

Investigations after spinal operations on large patient populations show a concentration-dependent shift in the microbial spectrum in the case of an infection after application of intrawound vancomycin powder. Gram-negative bacteria were dominant in patients in whom 1 g vancomycin powder was used, whereas gram-positive bacteria were dominant in those in whom 2 g powder was used^24^. Even if the mechanism is unclear, a selective pressure of locally applied vancomycin cannot be ruled out.

The minimal inhibitory concentration of vancomycin on methicillin-resistant Staphylococcus aureus (MRSA), Staphylococcus epidermidis, Haemophilus influenzae, Pseudomonas aeruginosa, Burkholderia cepacia, and Escherichia coli was determined to be 1.6 µg/ml, so that 5 µg/ml were enough to kill all bacteria within one day after exposure in vitro^26^. This is supported by the guidelines of the infectious disease society of America, that recommend serum vancomycin concentrations > 10 µg/ml to avoid the development of resistance and 15–20 µg/ml for a sufficient treatment^27^. In vivo, 2 g of intrawound vancomycin powder resulted in wound concentrations of 207 µg/ml one day after operation^26^^,^ so that therapeutic concentrations are to be expected even under unfavorable conditions. At the same time, a maximum serum concentration of less than 5 µg/ml is achieved 12 h after the operation, so that systemic side effects are unlikely^28^.

Vancomycin does not show any penetration of biofilm, so that it may only be used in prevention of infection or in combination with antibiotics that act against biofilm.

The main limitation of the present study is its retrospective study design, which, even if all potential influencing factors remain constant, cannot exclude unknown confounders. In addition, not all patients were systematically followed up with regard to the endpoint. Those patients who returned to follow-up or were readmitted at their own wish were evaluated according to the MSIS criteria on the basis of the available data. Therefore, it cannot be excluded that infected patients that regretted treatment in our hospital were not included in the analysis. However, it would appear plausible that the rate of such patients does not differ between the groups, so that the group differences and thus the conclusions remain unchanged.

The main advantage of the study, despite its monocentric design, is the high number of cases included in a short period of time, which potentially decreases the chance of confounding variables. In contrast to multicentric studies or investigations with a long inclusion period, this meant that influencing factors relevant to periprosthetic joint infection (preoperative antiseptic washing, use of drapes, clipping, type of single-shot prophylaxis and of antiseptic for washing, dressing, etc.) could be kept constant, so that the use of intraarticular vancomycin remains as the only variable.

Until data concerning the effect of vancomycin on cartilage are available, its use in unicompartmental knee arthroplasty or hemiarthroplasty of the hip must be viewed critically. None of the infected cases in the vancomycin group in our study showed resistance against vancomycin. Nevertheless it is worrying to use vancomycin as an antibiotic of last resort for infection prophylaxis. Local application of vancomycin in joints is not approved and therefore off-label. The patients should therefore sign an informed consent prior to operation.

On the basis of the results of the present study, intraarticular application of vancomycin powder in total hip and knee arthroplasty may be considered. Prospective randomized studies have to confirm this promising results prior a common recommendation.
